# Supplementary figures and images for: SuperFreq: Integrated mutation detection and clonal tracking in cancer
Source: PLoS Comput Biol. 2020 Feb 13;16(2):e1007603. doi: 10.1371/journal.pcbi.1007603 (PMC7043783; doi:10.1371/journal.pcbi.1007603)

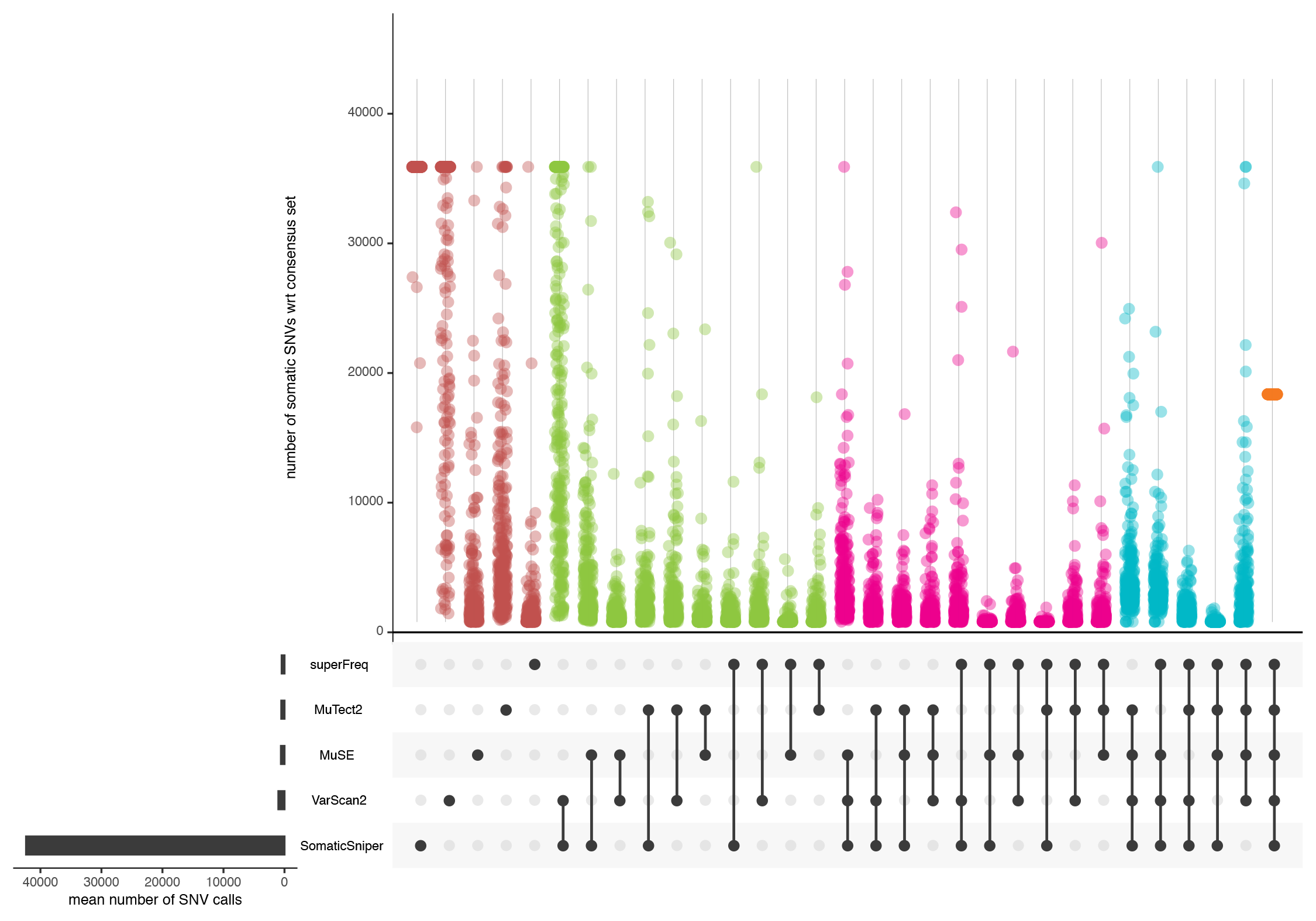

Supplement: S1 Fig — The fraction of SNVs called by subsets of 5 different SNV callers, relative to the number of SNVs called by all callers. Participants with less than 10 SNVs called by all methods are not included. The fraction is capped at 2. Graphics produced with the help of UpSetR. (TIF) [file pcbi.1007603.s001.tif]

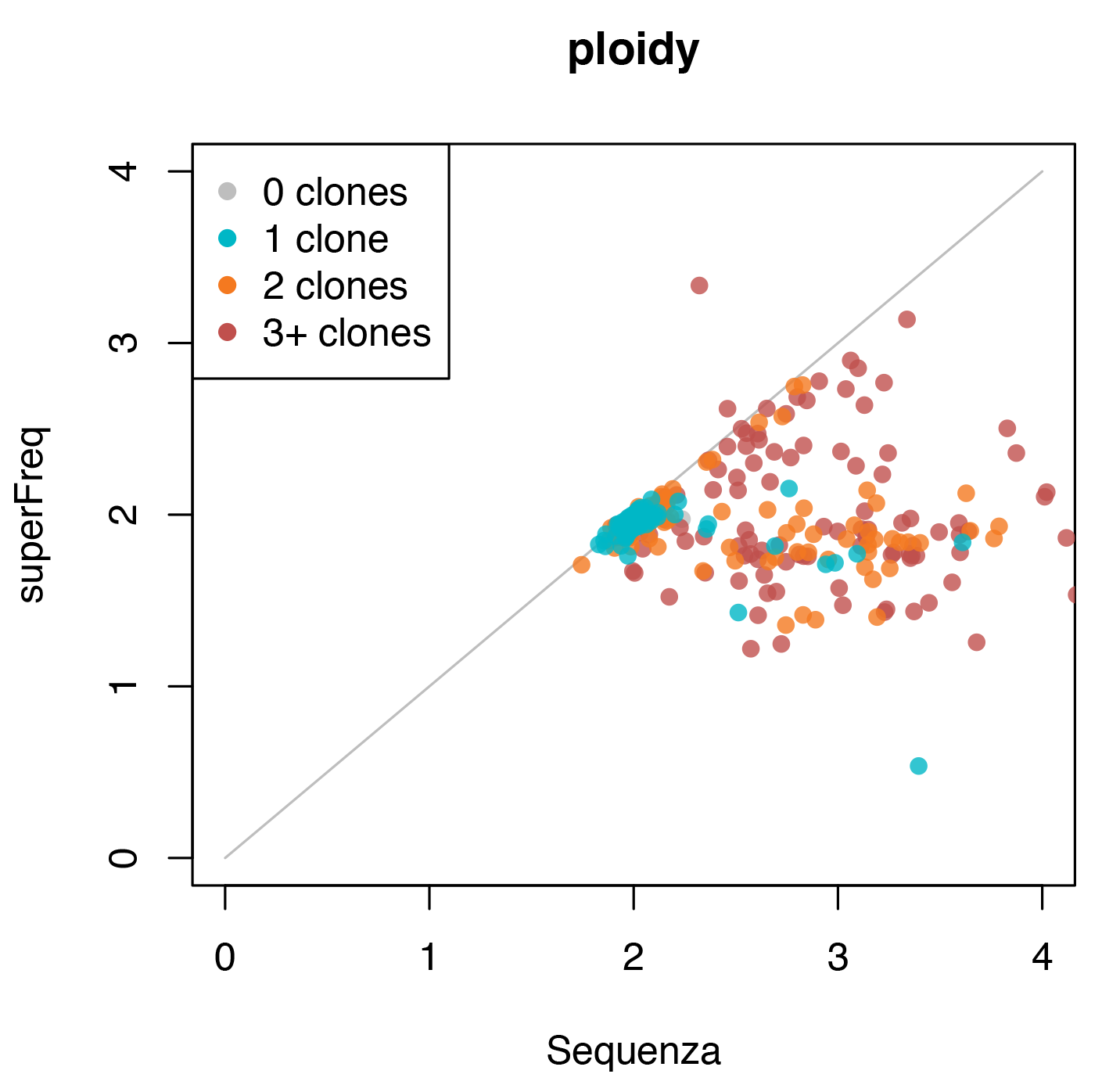

Supplement: S2 Fig — Ploidy calls from Sequenza and SuperFreq were coloured based on the number of cancer clones called by SuperFreq. (TIF) [file pcbi.1007603.s002.tif]

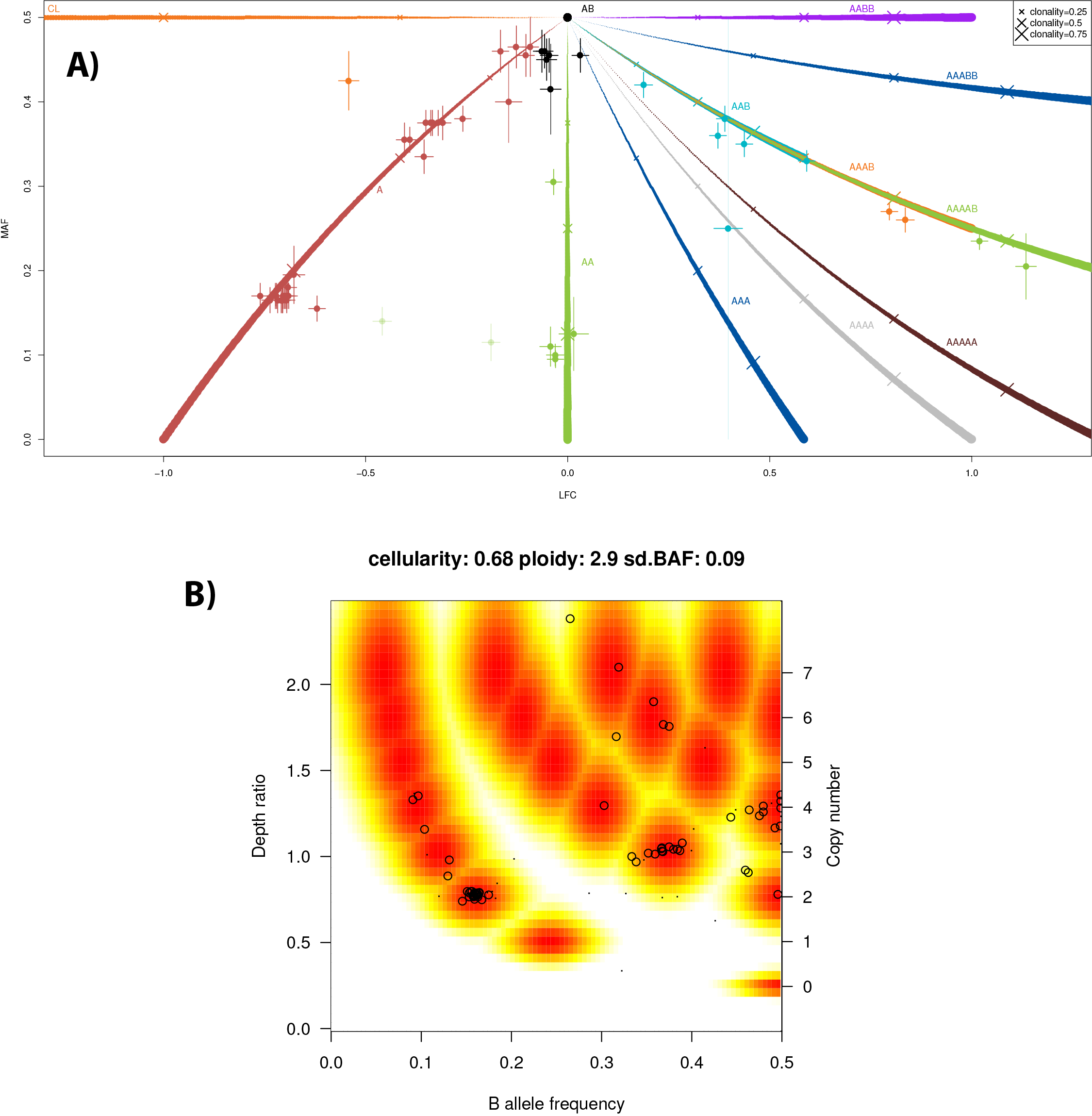

Supplement: S3 Fig — (A) Maypole plot showing the model fit for the ploidy call (or equivalently LFC normalisation) in SuperFreq. Coloured lines show expected LFC and MAF of different copy number calls, with lines growing thicker with the clonality of the call. Dots show data from each segment with uncertainty in LFC and MAF, allowing for heteroscedasticity. Normalisation corresponds to a constant shift along the x-axis to make the crosses fit with the lines within errors. (B) Sequenza model fit of the ploidy and purity call. Linear copy number call on the y-axis roughly corresponds to the x-axis of the maypole plot, and the x-axis in the Sequenza plot is the y-axis of the maypole plot. The single purity gives rise to single points for each copy number call, and the uncertainty is shown for the expected call rather than for the data points. (TIF) [file pcbi.1007603.s003.tif]

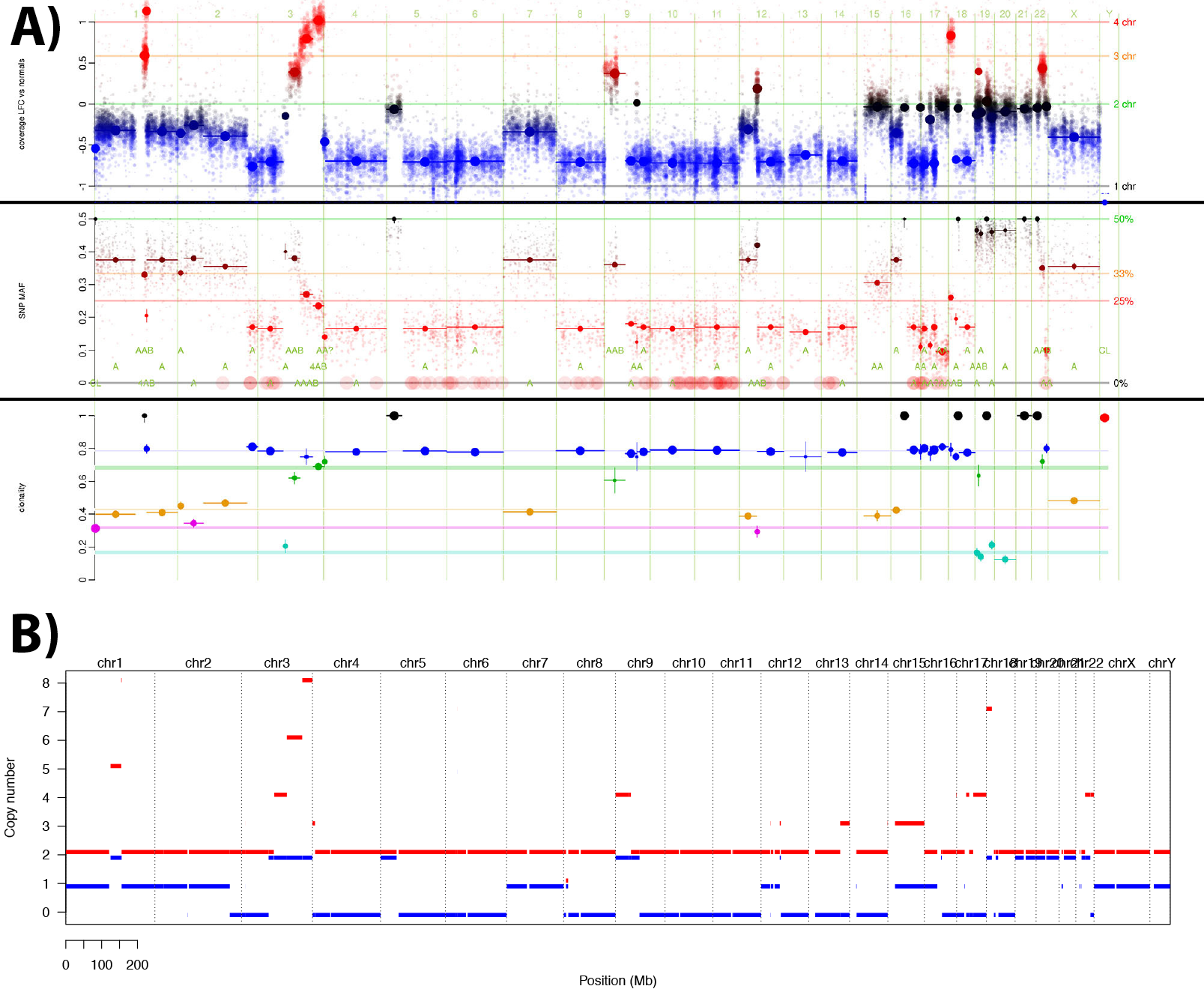

Supplement: S4 Fig — (A) Copy number calls from SuperFreq, showing LFC, MAF and clonality of the call. The size of the dots represent accuracy, based on the adjusted limma estimates for LFC, and based on the effective coverage for the BAFs. Segments, shown as dots with horizontal lines, also shows error estimates through an error bar and point size, and the extension of the segment on the x-axis. CNA calls are shown below the BAF segments, where uncertain calls (inconsistent data) are marked with "?" or "??". (B) Copy number profile from Sequenza with a purity of 0.68. Red shows major copy number, blue shows minor allele copy number. (TIF) [file pcbi.1007603.s004.tif]

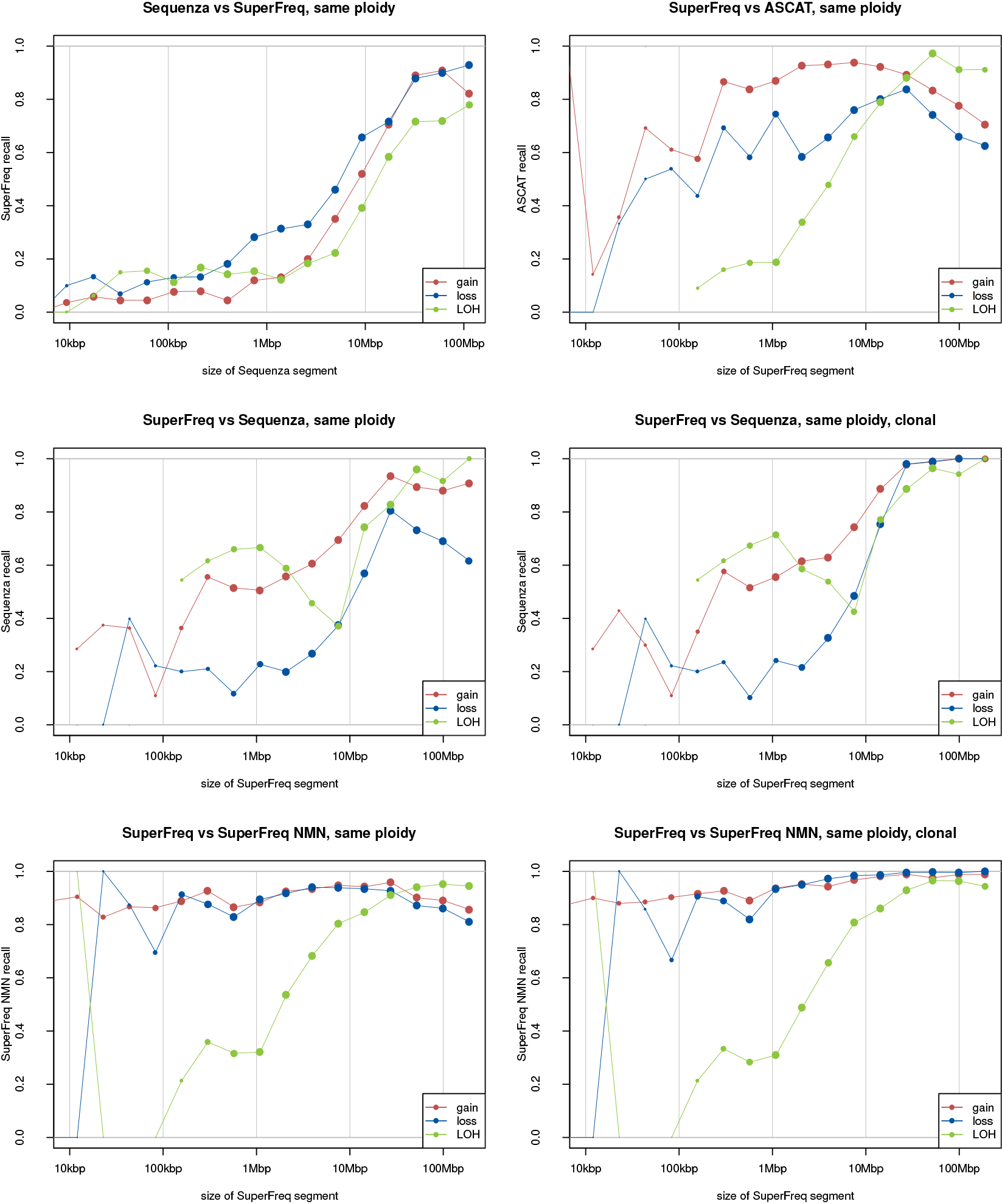

Supplement: S5 Fig — Recall of gain, loss and CNN-LOH binned by size of the segment, limited to participants where the ploidy agrees within 0.2 between the methods. "Clonal" indicates that the truth segments are limited to CNAs where SuperFreq called a clonality above 0.5. (TIF) [file pcbi.1007603.s005.tif]

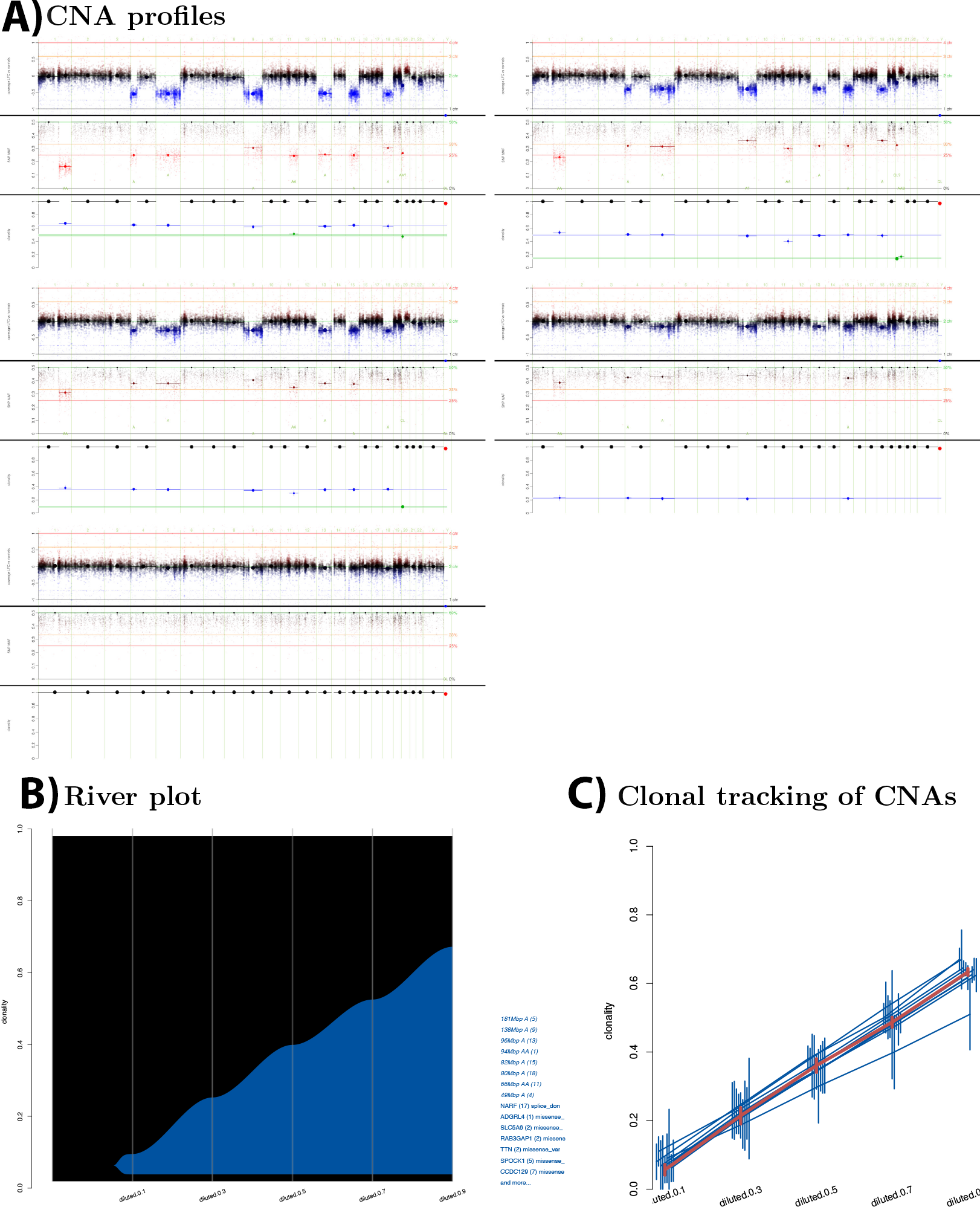

Supplement: S6 Fig — First panels are copy number calls at dilutions of 0.9, 0.7, 0.5, 0.3 and 0.1. Last three panels show the clonal tracking: river plot (germline variants removed) and line plots across the dilutions. Although some CNAs are not called at the 0.3 dilution, and none is called at 0.1, they are still tracked and are assign accurate clonalities as shown in the last panel (not default SuperFreq output) where tracked CNAs are shown as blue lines, and the clone is shown in red. SuperFreq shares the call and the segment coordinates across samples and queries the clonality by forcing the copy number call onto the segment in the other samples. In case the CNA is truly not present, the confidence interval is expected to overlap a clonality of 0. This analysis is performed without the matched normal sample, as the matched normal was used to dilute the cancer sample. (TIF) [file pcbi.1007603.s006.tif]

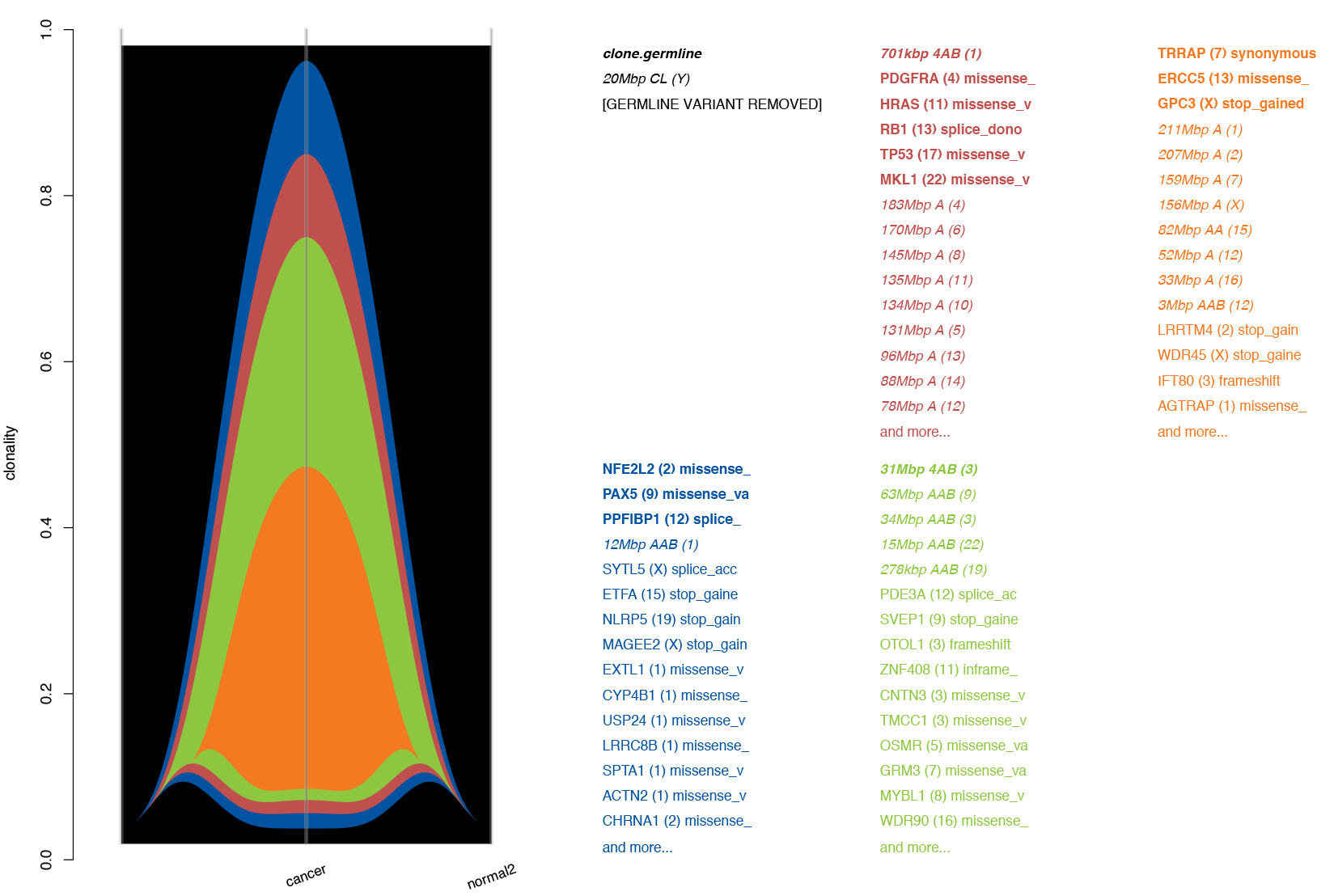

Supplement: S7 Fig — The original cancer has 4 clones called by SuperFreq. The copy number profile is shown in S4 Fig. (TIF) [file pcbi.1007603.s007.tif]

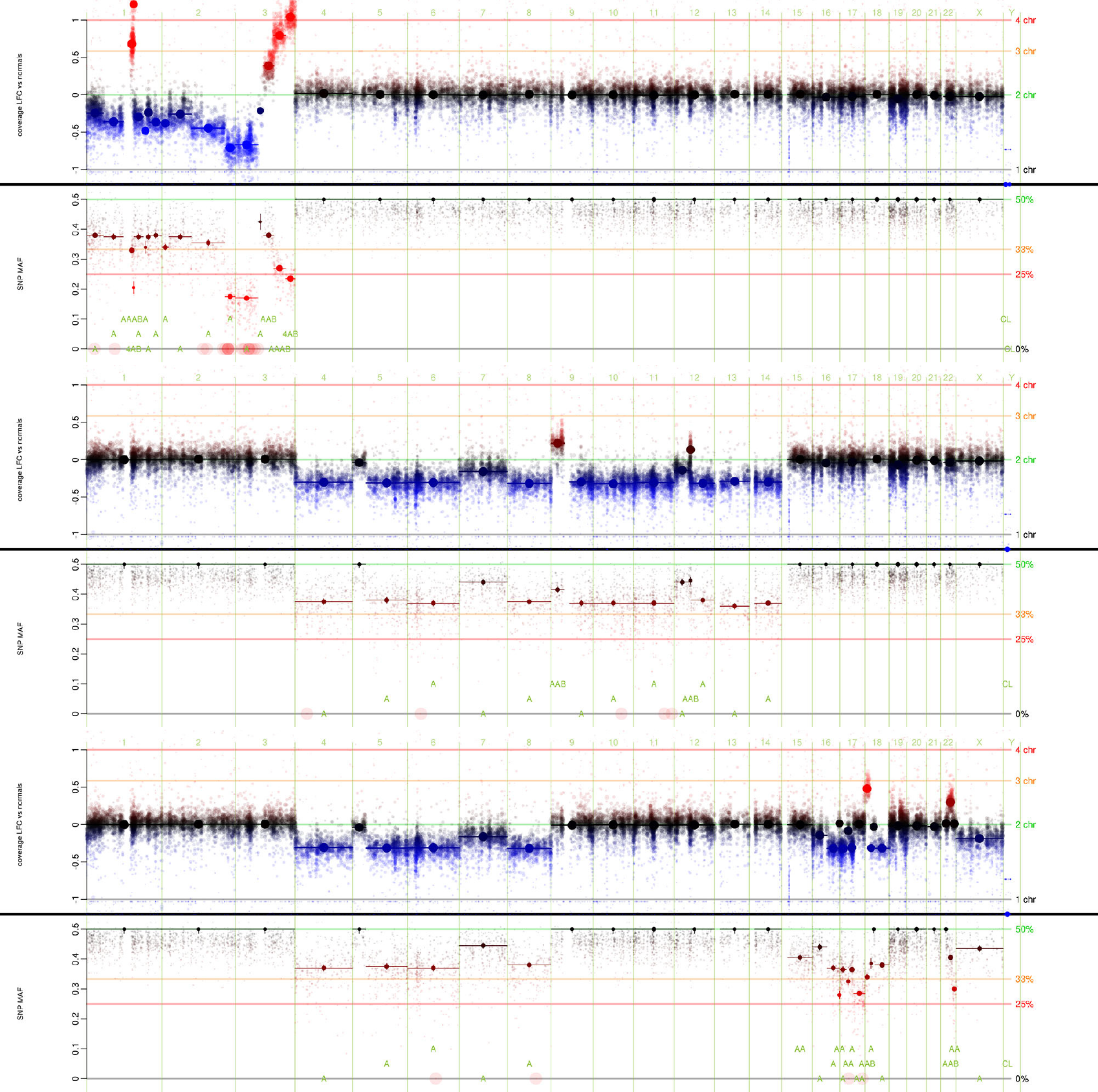

Supplement: S8 Fig — CNA calls over the genome showing LFC and BAF. The size of the dots represent accuracy, based on the adjusted limma estimates for LFC, and based on the effective coverage for the BAFs. Segments, shown as dots with horizontal lines, also shows error estimates through an error bar and point size, and the extension of the segment on the x-axis. CNA calls are shown below the BAF segments, where uncertain calls (inconsistent data) are marked with "?" or "??". The three simulated samples draw from mutations in different subsets of the chromosomes and of different admixtures of normal and cancer samples as illustrated by the copy number calls of the three samples. This process is described in Fig 4A in the main paper. The copy number profile of the original cancer is shown in S4 Fig. (TIF) [file pcbi.1007603.s008.tif]

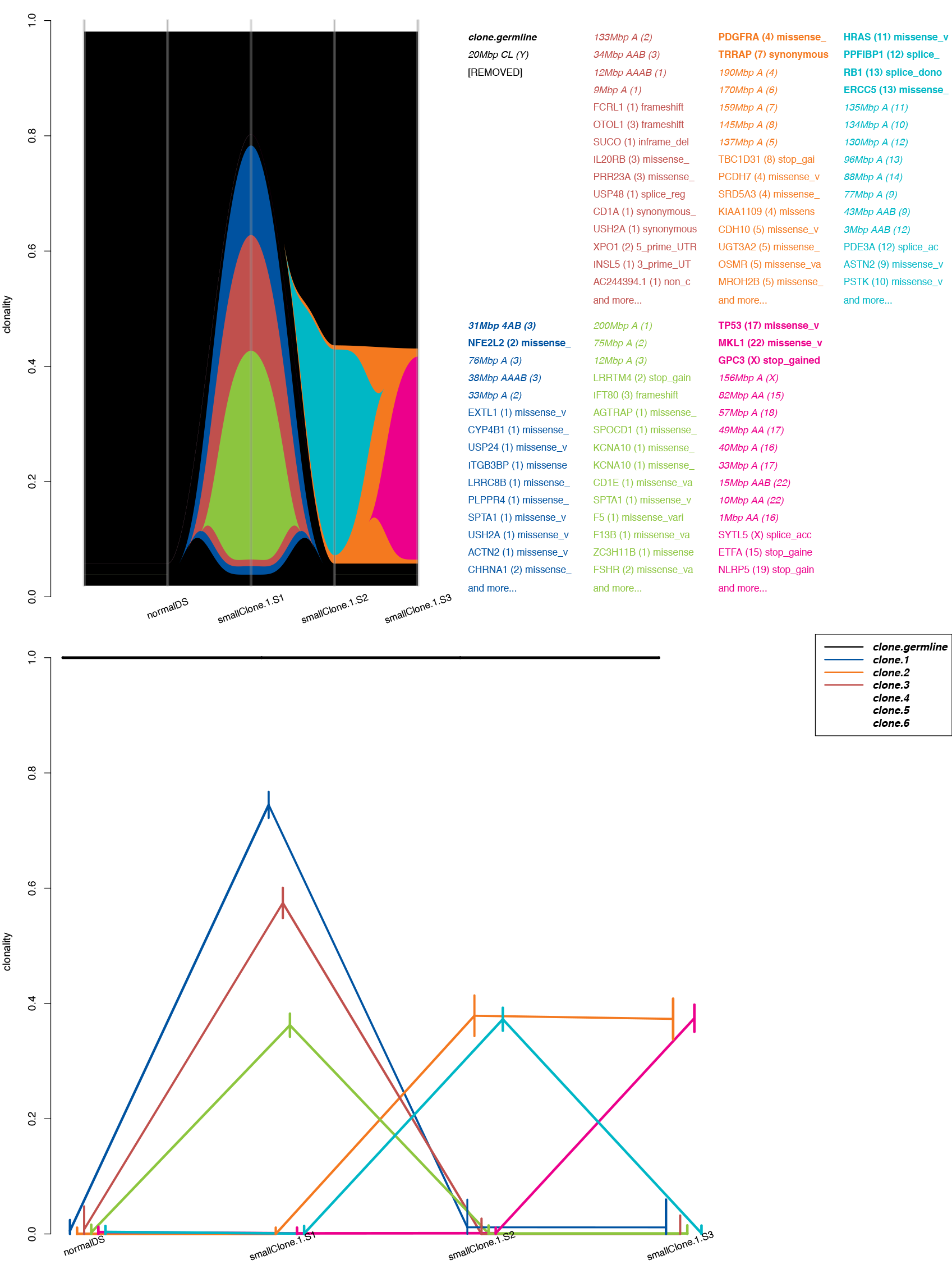

Supplement: S9 Fig — The SuperFreq clonal tracking of the simulated samples detects 3 subclones of the first clone based on the mutations on chr1 to chr3, while subclones are not detected for the other clones. We see that the mutations listed in each clone are found on the expected chromosomes from the schematic in Fig 4A in the main paper. (TIF) [file pcbi.1007603.s009.tif]
