# Supplementary material for: SuperFreq: Integrated mutation detection and clonal tracking in cancer
Source: PLoS Comput Biol. 2020 Feb 13;16(2):e1007603. doi: 10.1371/journal.pcbi.1007603 (PMC7043783; doi:10.1371/journal.pcbi.1007603)
Supplement: S1 Table — truth: Total number of coding somatic variants called in SuperFreq with matched normal. TP: Number of coding variants recalled (True Positives) without a matched normal (total 91%). FNP: Fraction of lost coding variants (False Negatives) without matched normal that are present in Population databases dbSNP or ExAC (100%). FP: Number of coding variants called without matched normal, not called with a matched normal (False Positives). TPg: Number of variants recalled (True Positives) without a matched normal after filtering on the germlineLike flag (total 87%). FPg: Number of coding variants called without a matched normal after germlineLike filter, not called with a matched normal. FPgP: Number of coding variants called without a matched normal after germlineLike filter, not called with a matched normal (False Positives), that are present in Population databases dbSNP or ExAC. We note that the number of false calls does not seem to depend on the number of true mutations, which confirms that the absolute number of false calls is a more robust measure of performance than normalised measures such as precision. (PDF) [file pcbi.1007603.s010.pdf]

	truth	TP	FN_P	FP	TP_g	FP_g	FP_{gP}
AML.080	296	273	23/23	230	271	58	30
AML.084	10	8	2/2	263	5	88	55
AML.102	9	7	2/2	216	3	76	47
AML.110	13	12	1/1	193	5	48	26
