# Supplementary material for: SuperFreq: Integrated mutation detection and clonal tracking in cancer
Source: PLoS Comput Biol. 2020 Feb 13;16(2):e1007603. doi: 10.1371/journal.pcbi.1007603 (PMC7043783; doi:10.1371/journal.pcbi.1007603)
Supplement: S2 Table — Recall, fraction false negatives in dbSNP, number of false positive somatic variants and runtime with 4 cpus in AML.084 with 5, 3 and 2 reference normals, using the analysis with 10 reference normals as truth. The runtimes are for the first run with the reference normals, subsequent runs of other samples using the same reference normals reuse gene counts and variants which decreases runtime. In our experience, the quality of the reference normals in mimicking the studied samples biases is more important than the number of reference normal samples. (PDF) [file pcbi.1007603.s011.pdf]

AML.084	Recall	FN in dbSNP	FP	runtime
10 normals	69/69	0/0	0	150m
5 normals	65/69	3/4	12	129m
3 normals	63/69	5/6	12	119m
2 normals	66/69	2/3	21	106m
